# Validation of a short food group questionnaire to determine intakes from healthy and unhealthy food groups in 5–9‐year‐old South African children

**DOI:** 10.1111/jhn.13249

**Published:** 2023-10-05

**Authors:** H Salome Kruger, Persuade Makore, Tertia van Zyl, Mieke Faber, Lisa J. Ware, Makama A. Monyeki, Ruan Kruger

**Affiliations:** ^1^ Centre of Excellence for Nutrition North‐West University Potchefstroom South Africa; ^2^ Medical Research Council Research Unit for Hypertension and Cardiovascular Disease North‐West University Potchefstroom South Africa; ^3^ Non‐Communicable Diseases Research Unit South African Medical Research Council Cape Town South Africa; ^4^ SA MRC‐Wits Developmental Pathways for Health Research Unit University of the Witwatersrand Johannesburg South Africa; ^5^ Physical Activity, Sport and Recreation Research Focus Area North‐West University Potchefstroom South Africa; ^6^ Centre of Excellence: Hypertension in Africa Research Team North‐West University Potchefstroom South Africa

**Keywords:** dietary intake, healthy foods, questionnaire, unhealthy foods

## Abstract

**Background:**

Reliable dietary data for children are necessary to investigate associations with health outcomes. The present study aimed to develop and validate a questionnaire to determine the frequency of intakes of specific healthy and unhealthy food groups in young children.

**Methods:**

Participants were 5–9‐year‐old South African children (*n* = 920) from 10 urban schools. Their parents completed a demographic questionnaire and the food intake questionnaire with food pictures. Based on the literature, four healthy food groups (fruits, vegetables, milk, meat/fish/poultry/eggs) and six unhealthy food groups (hot and cold sugar‐sweetened beverages, candy, salty snacks, cakes and fast foods) were included, with five different frequency responses. Six experienced nutritionists assessed the face validity and content validity. After pilot testing, construct validity and homogeneity were determined in the participants. Convergent validity was determined using urinary sodium and potassium concentrations as biological intake markers.

**Results:**

Nutritionists confirmed face and content validity. Caregivers confirmed understanding of the questionnaire. Three factors explained 50.2% of the variance, with most unhealthy food groups as factor 1, fruits and vegetables as factor 2, and animal source protein and milk groups clustered with sugar‐sweetened beverages as factor 3. The frequency of milk group, fruits and vegetables intake correlated negatively, whereas the frequency of salty snacks and fast foods intakes correlated positively with the urinary sodium:potassium ratio.

**Conclusions:**

The healthy and unhealthy food group questionnaire has advantages of low respondent burden, as well as acceptable content and convergent validity in South African children. The questionnaire may be used to investigate associations between food intakes and health outcomes.

## Key points

- Simplified methods to collect data on the food consumption of children are required in epidemiological studies.
- Intakes from specific food groups, namely fruits, vegetables, sugar‐sweetened beverages and snacks, salty snacks, and fast foods, are important.
- A healthy and unhealthy food group questionnaire was validated and showed acceptable content and convergent validity in South African children.
- The food group questionnaire has a low respondent burden.

## Graphical abstract

The study aimed to develop and validate a questionnaire to determine the frequency of intake of healthy and unhealthy foods in young South African children. The food group questionnaire had low respondent burden, acceptable content and convergent validity and can be used to study associations between food intakes and health outcomes.

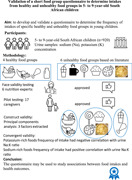

## INTRODUCTION

Globally, one in five children and adolescents are overweight.^1^ The rise in overweight and obesity, especially in Africa, is not unique to urban settings and also expands to rural environments because of increasing access to energy dense and processed foods, as well as increasing screen and sedentary time or decreased energy expenditure.^2^ The most recent data for 5–9‐year‐old South African children revealed a prevalence of 15% overweight and 4% obesity.^3^ Unhealthy dietary behaviours in late childhood and adolescence, as well as concomitant obesity, may result in lifelong adversity and an increased risk for non‐communicable disease and early onset cardiovascular disease. Overweight and obesity remain the most important determinants of childhood onset essential hypertension,^4^ which is linked to excess sodium intake, whereas less is known about the role of low potassium intake.^5^

Adopting healthy lifestyle habits such as healthy eating could lower the risk of becoming obese and developing related diseases.^6^ Food intake throughout childhood is an influencing factor of child growth and development, and energy‐dense diets are linked to obesity.^7^ Intake of three or more servings of sugar‐sweetened beverages (SSBs) per day was shown to be associated with overweight and obesity in Australian schoolchildren, and was identified as a target for intervention programmes aimed at children.^8^ Food preference learning starts during infancy and remains relatively stable during childhood years, but can be modified over time, although some original food habits persist, and are reflected in food choices that are made later in life.^9^ Interactions between the food environment and children's food preferences are key in identifying the factors that can be modified in order to improve healthy eating habits and prevent unhealthy weight gain in children.^8^, ^9^

Collecting reliable data on the dietary intake of children and the contributions of dietary intake to childhood nutritional status is a challenge.^10^, ^11^ The relationship between different types of foods and childhood obesity and obesity‐related conditions, however, should be investigated to contribute information to inform interventions.^12^ In South African studies, the dietary intakes of primary school‐age children are mostly collected by means of a 24‐h recall conducted with the aid of the caregiver of the child.^13^, ^14^ This method presents logistical challenges in studies with a large sample size because most often data are collected at the schools, where the caregiver can seldom be present. Therefore, simplified methods to collect data on the types of foods children eat, and in particular foods rich in selected nutrients, are required in epidemiological studies. Dietary intake data collected by questionnaire are subjective and it is important to find clinically useful ways to identify excessive or suboptimal intakes of foods rich in selected nutrients, such as sodium and potassium in children. Nutritional biomarkers provide an objective measure of dietary intake, independently of the errors inherent from self‐reported dietary recall methods.^15^

Foods may be grouped according to their nutrient profile and energy density.^10^ In the World Health Organization (WH)O Global school‐based student health survey (GSHS), a questionnaire that focussed on four food groups, namely fruits, vegetables, carbonated soft drinks and fast foods, was initially used.^16^ This questionnaire was later expanded to include different food groups, including milk products, high‐fat foods and salty foods.^17^ Similar simplified questionnaires have been used to determine the frequency of intakes of specific food groups in relation to the nutritional status of school‐age children.^8^, ^18^, ^19^, ^20^

The present study is part of a larger study aimed to investigate the impact of lifestyle behaviours including physical activity, dietary intake and psychosocial factors involved in early vascular compromise among South African children.^21^ The aim of this part of the study was to develop and validate a questionnaire to determine the frequency of intakes of specific healthy and unhealthy food groups in primary school‐age children. This instrument will then be used in further studies to examine the relationship between the frequency of intakes of specific healthy and unhealthy food groups with adiposity related outcomes among school‐age children.

## METHODS

This study is affiliated to the longitudinal Exercise, Arterial Modulation and Nutrition in Youth South Africa (ExAMIN Youth SA) study among 5–9‐year‐old children (*n* = 1062).^3^, ^21^ The study protocol was reviewed and approved by the Health Research Ethics Committee of the North‐West University (NWU‐00091‐16‐A1) and registered in a clinical trials registry (ClinicalTrials.gov Identifier: NCT04056377), following the Standard Protocol Items: Recommendations for Interventional Trials (SPIRIT) guidelines.^22^ This part of the study has a cross‐sectional design and only baseline data of the longitudinal study are included.

### Sample size calculation

Principals from urban schools from the Dr Kenneth Kaunda district were approached and invited to participate in the ExAMIN Youth SA study. Approval was obtained from 10 schools where baseline data were collected from 1103 children over a period of 19 months. Based on the original sample size calculation of approximately 1000 children, and after accounting for dropouts or school absentees, 1062 children with complete data were included with an achieved statistical power of 95.3% for the ExAMIN Youth SA study.^21^

### Population and setting

In 2017, the research team sought permission from the district Department of Education and school principals from all public schools within the Dr Kenneth Kaunda district in the North West province, South Africa, for participation in this research project. In South Africa, public schools are categorised into five quintile groups based on the employment rate and literacy of the community in which the school is located, as a measure to determine government funding of schools. Quintile 1 represents the poorest schools and quintile 5 represents the most affluent.^23^ From two municipalities, 50 km apart, principals of five schools from quintile 3 and five schools from quintiles 4–5 agreed to participate.^3^, ^21^

Children between the ages of 5 and 9 years from both sexes and all ethnic groups were recruited from the participating schools. With parental consent, 1103 children were invited to participate in the study during 2017–2019. No strict exclusion criteria were applied, but children who showed symptoms of cold, flu or fatigue on the day of measurement were not screened. In addition to parental consent, each child signed an informed assent (younger than 7 years) or a consent form (7 years and older) before any measurements were taken. In total, 1062 children (485 boys and 577 girls) were included in the final analysis at baseline, after excluding children with missing or incomplete data (*n* = 41). From the food intake questionnaires, 14 had to be excluded due to incomplete or implausible responses. For the main analysis of this study, we obtained complete data of a total number of 1039 healthy children, and complete data from the food intake questionnaire from 920 children.

Baseline data collection took place at each school separately in accordance with the data collection schedule. A day before data collection, children received a General Health and Demographics Questionnaire, the food intake questionnaire, and an information sheet on the measurements to take home. The General Health and Demographics Questionnaire included data on personal and family information (education of the parents, employment, type of dwelling and household income) and self‐reported health status.^21^ Parents had to complete the questionnaires and return them on the day of participation, as reported in detail elsewhere.^21^

Anthropometric measures were performed by trained postgraduate Physiology and Human Movement Sciences students according to the International Society for the Advancement of Kinanthropometry (ISAK) protocol.^24^ Body weight was taken without shoes and with the child dressed in light clothing with a model 813 digital scale (Seca) to the nearest 0.1 kg. Height was measured barefoot to the nearest 0.1 cm with a model 213 stadiometer (Seca) with a perpendicular board. All measurements were repeated at least twice and were recorded. The average of the two closest measurements was calculated and used in data analysis. We calculated body mass index (BMI) by dividing weight in kilograms by height in meter squared (kg/m^2^). The WHO 2006 growth software (Anthroplus) was used to calculate the height‐for‐age and BMI‐for‐age *z*‐scores (BAZ)^25^, ^26^ for the identification of underweight (BAZ < −2), normal weight (BAZ −2 to 1), overweight (BAZ > 1 to 2) and obesity (BAZ > 2) according to the child growth reference for children aged 5–19 years old.^25^

### Food intake questionnaire

#### Development based on a review of evidence from the literature

Children's usual dietary intake from ten food groups was assessed using the simplified unquantified food frequency questionnaire that was developed for the present study based on evidence from the literature (Table 1). The steps in the development and validation of this questionnaire focused on validity and reliability.

### Content validity

The questionnaire was primarily based on the questionnaire that was used in the WHO Global school‐based student health survey (GSHS), which initially focussed on four food groups, namely fruits, vegetables, carbonated soft drinks and fast foods.^16^ For the present study, the questionnaire was then further developed based on questionnaires that were used to determine intakes of healthy and unhealthy foods of children from Africa, Australia and the USA, which listed two to five healthy food groups (fruits, vegetables, legumes, fish and meat), four to five unhealthy food groups (cold/soft drinks, cookies/cake, candies and fast foods) and five different responses of frequency of intake.^8^, ^18^, ^19^, ^20^, ^27^ Thereafter, the questionnaire was refined and finalised based on the questionnaires used in studies that were performed in South Africa.^18^, ^28^, ^29^ The food groups included in this questionnaire are foods generally eaten by South African school children,^28^, ^30^ and include four groups of healthy foods, namely fruits, vegetables, milk products and meat/fish/poultry/eggs, and six groups of unhealthy foods, namely hot sweetened beverages (tea and coffee), cold SSBs, candy, salty snacks, cakes and fast foods. The hot beverages group (tea and coffee) with sugar was included as an additional SSB, because a study in South Africa reported that tea was consumed more frequently than cold SSBs by children aged 5–13 years old.^28^ Five different responses of frequency of intake were used, namely never, 1–2 days, 3–4 days, 5–6 days or 7 days per week. The period of recall for this questionnaire was during the past 4 weeks. Healthy foods were defined as foods containing essential nutrients for child growth and general health, namely fruits, vegetables, milk and meat/fish/poultry/eggs.^18^ Unhealthy foods were defined as foods that provide energy, sugar, salt and fats, but do not make an important contribution to essential nutrient intake.^16^

### Face validity and content validity testing

Face validity is based on the opinions of experts in the relevant field about the ability of an instrument to measure the intended concepts.^31^ Six South African nutrition scientists with experience in dietary assessment and who were not members of the research team assessed the draft questionnaire for face validity and content validity, aiming to determine whether the questionnaire covers the entire domain related to healthy and unhealthy food groups and whether it measures the concepts intended. They approved the food groups included, but recommended that processed meat should be added to the fast‐food group because children from low‐income household frequently consume processed meats as fast food.^28^, ^30^ This recommendation was implemented in the final questionnaire used in the study (Supporting Information S1).

### Pilot test for comprehension

The questionnaire was pilot tested for comprehension in a group of 17 caregivers of 5–9‐year‐old children. The questionnaire was presented to the group individually with the request that they should read and complete the questionnaire and report back on the ease of reading, understanding and completion of each question, as well as the time necessary to complete the questionnaire. A coloured picture file with example pictures of foods from each group was presented with the questionnaire to facilitate responses (Supporting Information S2). This questionnaire was taken home to be completed with the assistance of the parent or caregiver and the child involved.

### Construct validity

The extent to which the questionnaire measures the intended construct and homogeneity was determined by principal components analysis to determine whether the instrument measures homogeneous constructs.^31^

### Convergent validity: Determining the association between the frequency of intake from selected groups and biological markers of intake

There are few gold standards in nutrition research and the true value for intake is seldom known. Furthermore, validation studies have confirmed important measurement errors inherent to self‐report methods.^11^ Therefore, a biological marker as proxy of true intake of sodium and potassium was used to determine convergent validity, which is an indication of whether values obtained from an instrument are highly correlated with similar variables obtained from other instruments.^31^ Based on an earlier study, children who consumed potassium‐rich fruit and vegetables daily had higher urinary potassium concentrations than those with lower intakes,^32^ whereas another study provided evidence that urinary sodium concentrations provide useful estimates of sodium intakes in children.^33^ The urinary sodium and potassium concentrations were measured from a spot urine sample, collected on the morning when children were measured at their schools and a urinary sodium:potassium (Na:K) molar ratio was calculated to capture both concentrations in one variable. The final step in the validation was to determine the association of the frequency of intake for milk, fruits and vegetables (foods with a high potassium content) with urinary Na:K molar ratio of individual children, as well as to determine the association of the frequency of intake for salty snacks and fast foods (foods with a high sodium content) with the same marker. The selection of these food groups was based on a report on the top 10 food category contributors to potassium and sodium intake by age in the USA.^34^

### Reliability

Reliability was tested by Cronbach's α calculation, a commonly used test to determine the internal consistency of an instrument. Test–retest reliability, also referred to as reproducibility, refers to an instrument's ability to produce consistent results with repeated measurements.^31^ Measurement error from repeated tests is not independent because both reports may be prone to reporting error. Interpretation of the test–retest reliability of questionnaires also present challenges, given that it captures different time periods for which dietary patterns could reasonably be different as a result of intra‐individual variability in intakes across a 4‐week period.^11^ For these reasons, and also because the respondent burden was already high, this questionnaire was not tested for reproducibility given the number of questionnaires parents had to complete^21^.

### Urine sampling and analysis

Instructions for urine collection and consumables for urine collection were given to each child to take home after school on the day before measurements were taken. The participants collected a first voided morning midstream urine sample at home and placed the urine in a cooling container. Urine samples were collected at the schools and delivered to the laboratory for preparation according to standard procedures and stored at −80°C prior to analysis. The ion‐selective electrode (ISE) module of the Cobas Integra system was used to quantify sodium and potassium in diluted urine using ion‐selective electrodes (Cobas Integra 400 plus; Roche Diagnostics, Basel, Switzerland). An ISE makes use of the unique properties of an ion‐selective membrane to develop an electromotive force for the measurements of ions in solution. The selective membrane is in contact with both the test solution and an internal filling solution. Because of the selectivity of the membrane, only the ions to be measured contribute to the electromotive force. The complete measurement system for a particular ion includes the ISE, a reference electrode and electronic circuits to measure and process the electromotive force to give the test ion concentration (mmol/L). The traceability has been standardised against primary calibrators prepared gravimetrically from purified salts. The sodium:potassium molar ratio was calculated as sodium concentration divided by potassium concentration. The standard laboratory protocol was followed in the Hypertension Research Laboratory (North‐West University, Potchefstroom, South Africa) and calibration was performed every 5 h for the ISE module, after which quality control samples from Roche Diagnostics and Randox (third‐party quality controls) were used as an additional quality control procedure. All controls were within acceptable ranges after which samples were analysed. For reproducibility, coefficients of variation were determined for both sodium (2.16%–2.41%) and potassium (1.85%–2.48%), which were below 3%.

### Statistical analysis

Descriptive data of demographic information (age, education of caregivers, household income and home language), anthropometric information, urinary sodium and potassium, and frequency of intakes from food groups are presented. The distribution of data was checked for normality using the Kolmogorov–Smirnov test and Q‐Q plots. Descriptive statistics were reported using median and interquartile ranges for non‐normal data, the mean ± SD for normally distributed data, and numbers and percentages for categorical characteristics. The five different responses of frequency of intake, namely never, 1–2 days, 3–4 days, 5–6 days or 7 days per week were coded as 0, 1, 3, 5 and 7. Missing data were not imputed, and tests were performed for cases with complete data for each test.

Principal components analysis with varimax rotation was used to assess the extent to which the questionnaire measures the intended construct and homogeneity of the questionnaire items. Item‐to‐item correlation was used to determine the Cronbach's α as an indicator of internal consistency. To determine the association between frequency of intakes from food groups and urinary Na:K ratio, Spearman correlation coefficients were calculated because most variables deviated from the normal distribution. A multivariable linear regression model was used to analyse the association of the frequency of intake for fruits, vegetables, salty snacks and fast‐food intakes, respectively, with the urinary Na:K ratio, with adjustment for covariates (children's age, sex, household income and educational status of the caregiver). To determine any dose–response associations, the urine sodium and potassium concentrations of children were also compared across tertile groups of frequency of fruit and vegetable intakes, as well as salty snacks and fast‐food intakes, using one‐way analysis of variance with linear trend analysis. Analysis was performed using SPSS, version 28 (IBM Corp.).

### Ethical considerations

The researchers obtained approval and permission from the Department of Education and Health Research Ethics Committee of the North‐West University (NWU‐00091‐16‐A1) and registered in a clinical trials registry (ClinicalTrials.gov Identifier: NCT04056377) to conduct the study. The parents and caregivers were required to sign informed consent forms for the children, whereas children whose parents consented were asked to sign assent (children < 7 years) or consent forms (children ≥ 7 years) for their participation in the study after they were provided with information about the study and their role and rights as participants.

## RESULTS

Basic characteristics of the participants are presented in Table 2. Of the 1062 healthy participants, 54% were black (*n* = 576), 44% were white (*n* = 463) and 2% were of mixed race or Indian ethnicity (*n* = 23). The steps in the development and validation of the food intake questionnaire and results of each step are shown in Table 1. Based on a review of evidence from the literature, a questionnaire with 10 food groups was developed.^8^, ^18^, ^19^, ^20^, ^27^ Six South African nutrition experts in dietary assessment approved the questionnaire based on face validity, after their recommendation for the addition of processed meat in the fast‐food group was accepted. In the next step, 17 caregivers of 6–8‐year‐old children confirmed that all questions in the questionnaire was easy to understand and complete. The caregivers found the questionnaire easy to understand and complete within 4–7 min and reported no inability to understand the language used in the questionnaire.

**Table 1:** The steps in the development and validation of the food intake questionnaire.

Method	Action	Outcome	References
Validity			
Content validity	The extent to which a research instrument accurately measures all aspects of a construct		
Review of evidence from the literature, step 1	Identify international questionnaire developed to estimate frequency of intake from healthy and unhealthy food groups	WHO Global school‐based student health survey questionnaire: four food groups	WHO, 2008^16^
Review of evidence from the literature, step 2	Further development based on regional studies in pre‐adolescent children.	Simplified food frequency questionnaire: 2–5 healthy food groups, 4–5 unhealthy food groups.	Sanigorski *et al*., 2006^8^
			Andaya *et al.*, 2011^19^
			Daboné *et al.*, 2013^18^
			Larsen *et al.*, 2015^20^
			Moreira *et al.*, 2015^27^
Review of evidence from the literature, step 3	Further development based on studies in South African children	Simplified food frequency questionnaire: four healthy food groups, six unhealthy food groups	Pedro *et al.*, 2008^28^
			Feeley *et al.*, 2012^30^
Face validity testing	Review by South African nutrition experts in dietary assessment to assess if the questionnaire covers the domains and measures the intended concepts	Processed meat added and specified in the fast‐food group. Confirm content validity and face validity	Pedro *et al.*, 2008^28^
			Feeley *et al.*, 2012^30^
			Heale & Twycross, 2015^31^
Pilot test for comprehension	Pilot tested for comprehension in 17 caregivers of 6–8‐year‐old children	Confirm that the questionnaire is easy to understand and complete	‐
Addition of visual examples	Improve comprehension of foods in each group by adding pictures	Coloured picture file with examples from food groups	‐
Construct validity	Factor analysis to assess if the instrument measures homogeneous constructs	Three factors: unhealthy foods (factor 1), healthy foods (factor 2) negative correlations with unhealthy foods, SSBs (factor 3)	Heale & Twycross, 2015^31^
Criterion validity, convergent validity	Spearman correlation: the frequency of intake for milk, fruits and vegetables respectively, with urinary Na:K ratio; the frequency of salty snacks and fast foods intake with urinary Na:K ratio. Multivariable linear regression with adjustment for age, sex, household income and educational status of the caregiver. Comparison of urine sodium and potassium concentrations across tertile groups of fruit, vegetable, salty snacks and fast‐food frequency of intake	Negative correlation between the frequency of fruits, vegetables and milk intake and urine Na;K ratio; Positive correlation between the frequency of fast food and salty snack intake and urine Na;K ratio. Fruit and vegetable groups: increasing frequency of intakes associated with increasing urine potassium concentrations; increasing frequency of intakes from the salty snacks and fast‐food groups associated with a trend of decreasing urine potassium concentrations	Woodruff *et al*., 2020^32^
Reliability			
Internal consistency	Cronbach's α is the most used test to determine the internal consistency of an instrument	Cronbach's α for all items = 0.51; for healthy food groups = 0.52; for unhealthy food groups = 0.58	Heale & Twycross, 2015^31^
Stability	Test–retest reliability: when an instrument is completed by the same participants more than once	Not performed due to high respondent burden, balanced with expected quality of outcomes	Tugault‐Lafleur, 2017^11^

**Table 2:** Descriptive characteristics of 5–9‐year‐old children (*n* = 1062).

Characteristic	Value
Age (years)	7.43 ± 0.91
Female sex, *n* (%)	577 (54.3)
Race, n (%)	
Black	576 (54.2)
White	463 (43.6)
Asian and mixed race	23 (2.2)
School quintile based on employment rate and literacy in the catchment living area, *n* (%)	
Quintile 3, no‐school fee schools, government funded	348 (32.8)
Quintile 4, fee‐paying schools, second highest income quintile	434 (40.9)
Quintile 5, fee‐paying schools, highest income quintile	278 (26.2)
Anthropometric data	
Body weight (kg)	25.0 ± 6.48
Body height (cm)	123 ± 7.82
BMI for age (*z*‐score)	0.03 ± 1.15
BMI for age *z*‐score categories, *n* (%)	
Underweight	40 (3.8)
Lean	814 (76.6)
Overweight	161 (15.2)
Obese	47 (4.4)

*Note*: Values are presented as the mean ± SD or number of participants and percentage.

Abbreviation: BMI, body mass index.

When the construct validity and homogeneity of the questionnaire were determined by principal components analysis, three factors were extracted. These factors explained 50.2% of the variance. Most unhealthy food groups, namely cookies, salty snacks, candy and fast food, clustered strongly together as factor 1, and cold SSBs also clustered with these groups, but it showed a stronger association with the animal source protein and milk groups (factor 3). The fruits and vegetables groups clustered strongly together as factor 2, with moderate associations with the animal source protein and milk groups (Table 3). Healthy foods tended to cluster in factor 2 and showed negative correlations with unhealthy food groups (SSBs, salty snacks and candy). Cronbach's α for all items was 0.51, reflecting a relatively low internal consistency of the questionnaire compared to the accepted value of 0.7 as an indicator of good internal consistency. Cronbach's α for healthy food groups was similar at 0.52, whereas Cronbach's α for unhealthy food groups was 0.58.

**Table 3:** Principal components analysis of the food intake questionnaire items.

Food groups	Factor 1	Factor 2	Factor 3^a^
Healthy food groups			
Fruits	0.075	**0.783**	−0.019
Vegetables	−0.051	**0.786**	0.029
Animal source protein	−0.047	0.347	**0.667**
Milk products	−0.119	0.307	**0.601**
Unhealthy food groups			
Sugar‐sweetened beverages	0.244	−0.221	**0.609**
Tea/coffee with sugar	0.075	−0.190	0.485
Cake, biscuits	**0.673**	0.063	0.077
Salty snacks, crisps	**0.738**	−0.070	0.045
Sweets, candy	**0.696**	−0.084	0.261
Fast foods	**0.610**	0.046	−0.188

*Note*: Component loadings >0.5 marked in bold show components clustering together.

^a^ The three factors explained 50.2% of the variance.

In assessment of the criterion validity of the questionnaire, weak negative correlations were found with urinary Na:K ratio and the frequency of intake for milk products (*r* = −0.14, *p* < 0.001), fruits (*r* = −0.09, *p* = 0.008) and vegetables (*r* = −0.09, *p* = 0.008), respectively. Furthermore, there were weak positive correlations between urinary Na:K ratio and the frequency of intake for salty snacks (*r* = 0.12, *p* < 0.001) and fast foods (*r* = 0.11, *p* = 0.001), respectively. A stronger negative correlation was found between household income and urinary Na:K ratio (*r* = −0.30, *p* < 0.001). In multivariate regression models, with adjustment for age, sex, household income and parental education level, there were no significant associations between frequency of intake from any food group with urinary Na:K ratio, and the variables explained a small percentage of the variance (data not shown).

**Figure 1:**
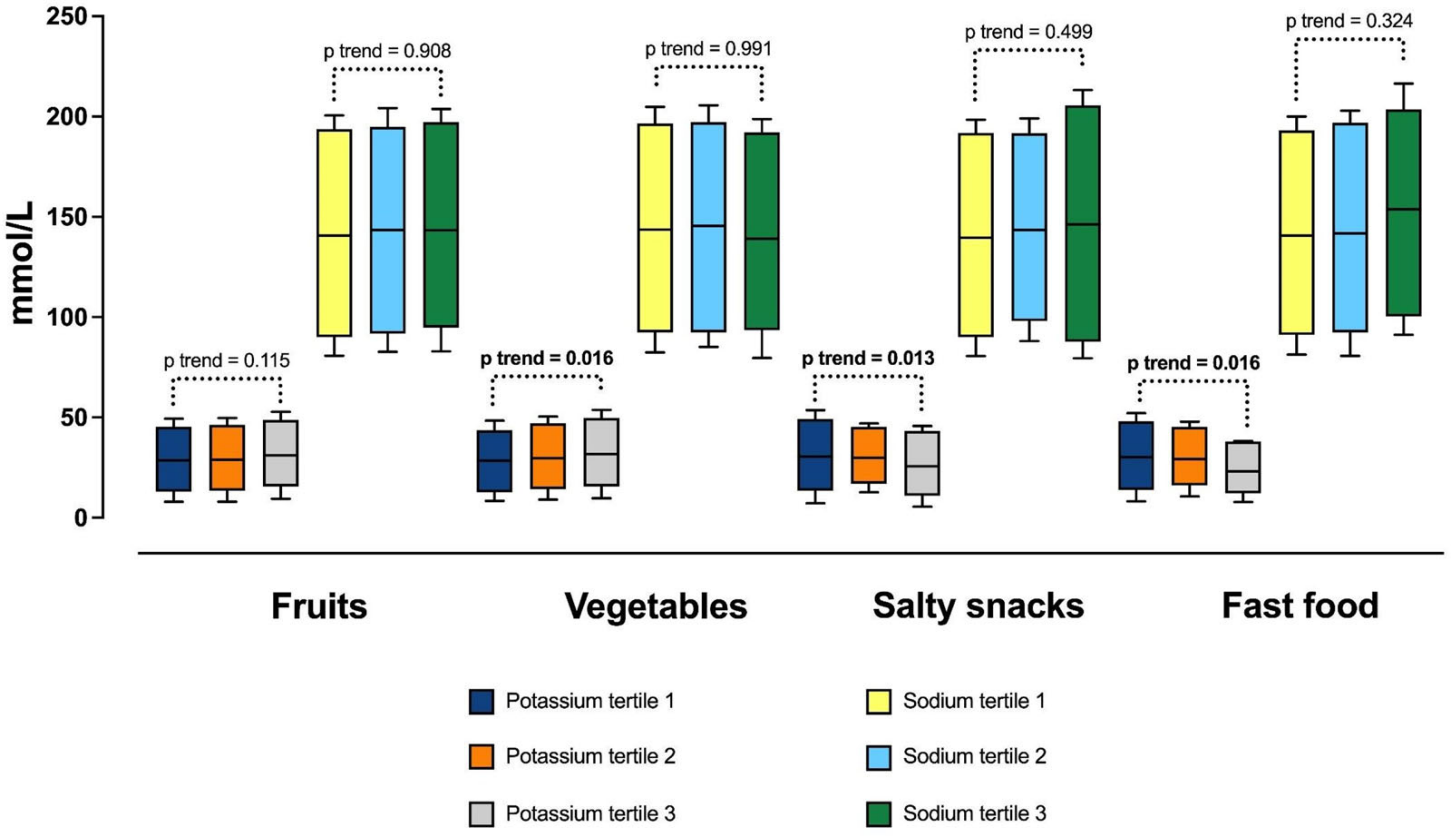
Comparison of urinary sodium and potassium concentrations of children across tertile groups pf frequency of fruits, vegetables, salty snacks and fast‐food intakes (median, interquartile range, minimum and maximum).

The urinary sodium and potassium concentrations of children across tertile groups of frequency of fruit and vegetable intakes, as well as salty snacks and fast‐food intakes, are shown in Figure 1. There were differences between the urinary potassium concentrations of the three tertile groups for three food groups, although these were in different directions. For the vegetable group, a higher frequency of intakes was associated with higher urinary potassium concentrations (*p* = 0.02), whereas higher frequency of intakes from the salty snacks (*p* = 0.01) and fast‐food groups (*p* = 0.02) were associated with lower urinary potassium concentrations. No significant differences between urine sodium concentrations were found across tertile groups for any of the food groups, although an apparent weak trend of higher sodium concentration across tertile groups of fast‐food intakes was observed (*p* = 0.32) (Figure 1).

## DISCUSSION

The main findings of the present study were that the food questionnaire had sufficient content validity and face validity to collect data on frequency of intake of healthy and unhealthy food groups by South African children aged 5–9 years old, and it was easy to understand and complete by caregivers of these children. To collect food intake data from such a young group is challenging, and therefore parents or caregivers provide information on dietary intakes in most cases. When data collection is performed at school, parents must complete the questionnaires at home and direct contact with the interviewers may not be possible. Despite these challenges, similar questionnaires have been used to determine the frequency of intakes of specific food groups of interest as determinants of healthy and unhealthy food patterns,^18^, ^19^, ^20^ or in association with obesity among school‐age children.^8^ The observed significant positive associations, although weak, between potassium‐rich and sodium‐rich food groups, respectively, and urinary Na:K ratios, may be an indication of the criterion validity of the instrument. Higher frequency of intakes from fruit and vegetable groups were associated with higher urinary potassium concentration and the frequency of intakes from the sodium‐rich fast‐food group tended to be positively associated with urinary sodium concentration.

According to the South African food based dietary guidelines children should consume vegetables and fruit frequently and use fats, salt and sugar sparingly.^35^ Insufficient intakes of fruit and vegetables have been reported among South African children, with possible health implications.^36^ Furthermore, frequent intakes of unhealthy snack foods and SSBs have been reported across household income groups.^28^, ^36^, ^37^ The dietary assessment tool most often used to assess adherence to the guidelines is the 24‐h recall, from which food items are then categorised in food groups.^14^ Collection of valid data with the 24‐h recall in children younger than 10 years is challenging,^10^, ^12^, ^14^ and therefore a shorter questionnaire focussing on food groups of interest in a particular study will be useful. Short, focussed questionnaires have been used in several studies to describe food patterns or to detect associations between intakes from healthy and unhealthy food groups and health outcomes.^8^, ^18^

The questionnaire items clustered within three factors, the first represents the unhealthy foods and with weak negative associations with vegetables, animal source protein and milk. The second factor represents the healthy food groups, with strong associations between fruit and vegetable groups, but moderate negative associations with SSBs, and weak negative associations with salty snacks and sweets. The third factor includes animal source protein, milk and SSBs, with a negative association with fast foods. SSBs are widely consumed, as previously reported in other South African studies among school‐age children,^28^, ^37^ and may therefore be associated with the animal source food pattern, but not with a food group pattern with the highest frequency of intake of fruit and vegetables. Principal component analysis indicates construct validity of the food group questionnaire, with sufficient ability to differentiate between food group patterns including mostly healthy and unhealthy foods. Although an indication of construct validity was confirmed, Cronbach's α for all items of 0.51 reflects a low internal consistency of the questionnaire compared to the proposed cut point of 0.7 for good internal consistency. The low Cronbach's α for healthy and unhealthy food groups is probably a result of the observation that the SSBs food group clustered more strongly with the animal source protein group than with unhealthy food groups.

The only biological markers for intake of particular nutrients of interest in the present study were urinary sodium and potassium concentrations, as markers of sodium and potassium intakes from food groups rich in these two nutrients. An early study suggested that 77% of potassium intake is excreted in the urine of adults.^38^ In a study where 24‐h urine samples were collected from 6–8‐year‐old children, those who consumed fruit and vegetables four or more times per day had higher urine potassium concentrations than the group with lower intakes.^39^ This may be an indication of a correlation between potassium‐rich foods (fruit and vegetables) and potassium excretion. A study in Chinese children showed that a spot urine sample had low predictive ability to estimate 24‐h urinary potassium concentrations.^39^ Studies on the validity of urinary sodium excretion as a marker of sodium intake are available. Although most studies were performed in adults,^40^, ^41^ one study provided evidence that urine spot samples may provide accurate estimates of the 24‐h sodium excretion in children.^33^ Because none of the prediction equations to estimate sodium intake from urine spot samples have been validated in South African children, we used a molar ratio of Na:K concentrations as the dependent variable to determine associations with frequency of intakes from food groups. Consistent findings from correlation analysis and comparison of sodium and potassium concentrations across tertile groups of frequency of intakes from sodium and potassium‐rich foods indicate that the questionnaire may be a valid tool to assess intakes of selected nutrients relevant to health.

The children included in the present study attended schools from the South African quintile groups 3–5, based on the employment rate and literacy of the community in which the school is located. Because quintile 1 represents the poorest schools and quintile 5 represents the most affluent,^23^ the participants of the study did not include those from the lowest socio‐economic status groups. The questionnaire may therefore not be appropriate to use in studies where most of the participants are from households with a high unemployment and low literacy rate. It is likely that parents from such households may not be able to complete the food group questionnaires without the direct assistance of a fieldworker. Furthermore children from these households may not eat a variety of foods from the fruits, milk, fast food and animal‐source protein groups included in this questionnaire and the frequencies may be too low to detect associations with health outcomes.^13^, ^14^, ^36^

### Limitations and strengths

Only a single morning midstream urine sample was available for analysis in the present study because collecting 24‐h samples from primary school‐aged children would be burdensome. Ideally, a 24‐h sample should be used to determine daily sodium and potassium excretion as a proxy for dietary intake. Estimated sodium excretion from spot urine samples may possibly be used to monitor trends in the population but is not recommended as an accurate measure of individual dietary intakes of sodium and potassium.^41^ This marker was the only available biological marker for use in the present study. Despite the noted limitations of the study, a strength was that it was the first to validate a short food group questionnaire to determine intakes from healthy and unhealthy food groups in a large sample of 5–9‐year‐old South African children. Additionally, the inclusion of urine sample biomarkers in the validation of the questionnaire is another strength of the study.

### Summary and conclusion

The limited availability of appropriately validated tools to quantify dietary intake from food groups of interest in association studies poses a challenge for the evaluation of intake of healthy and unhealthy foods among children. More methodological research is needed to improve the accuracy of self‐report instruments for assessing food intakes among school‐age children. A short food group questionnaire was developed and validated in the present study to determine intakes from healthy and unhealthy food groups in 5–9‐year‐old South African children. This instrument has advantages of low respondent burden, acceptable content and convergent validity, but also has limitations. The short food group questionnaire may be used to contribute evidence to evaluate the association between intakes from healthy and unhealthy food groups and health outcomes.

## AUTHOR CONTRIBUTIONS

RK is the Principal Investigator of the ExAMIN Youth SA study, responsible for the design and execution of the study, drafting of the protocol, securing funding, and ethics monitoring of the study progress. MAM is a senior co‐investigator of the study and lead participant recruitment and stakeholder reporting and supervised anthropometric data collection. RK supervised handling of urine samples and analyses. HSK, PM, TvZ and MF developed the South African specific healthy and unhealthy food intake questionnaire and collected data. All authors assisted in data analysis and drafting of this paper, read and approved the final version of the manuscript submitted for publication.

## CONFLICTS OF INTEREST STATEMENT

H Salome Kruger and M Faber are members of the Grant Review Panel for the South African Sugar Association. The other authors declare that they have no conflicts of interest.

### PEER REVIEW

The peer review history for this article is available at https://www.webofscience.com/api/gateway/wos/peer-review/10.1111/jhn.13249.

## ETHICAL STATEMENT

The study protocol was reviewed and approved by the Health Research Ethics Committee of the North‐West University (NWU‐00091‐16‐A1).

## TRANSPARENCY DECLARATION

The lead author affirms that this manuscript is an honest, accurate and transparent account of the study being reported. The reporting of this work is compliant with STROBE‐nut guidelines. The lead author affirms that no important aspects of the study have been omitted and there are no discrepancies from the study as planned (protocol and the registration identifiers).

## Supporting information

Supporting information.

Supporting information.

## Data Availability

Data for the study are not available as online material but are available from the authors upon reasonable request in accordance with the NWU policy guidelines.
