# Supplementary material for: Validation of a short food group questionnaire to determine intakes from healthy and unhealthy food groups in 5–9‐year‐old South African children
Source: J Hum Nutr Diet. 2023 Oct 5;37(1):234–45. doi: 10.1111/jhn.13249 (PMC10953415; doi:10.1111/jhn.13249)
Supplement: Supplementary file 1 — Supporting information. [file JHN-37-234-s002.docx]

Appendix S1: Food picture file with examples of foods from food groups.


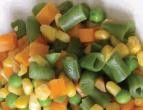


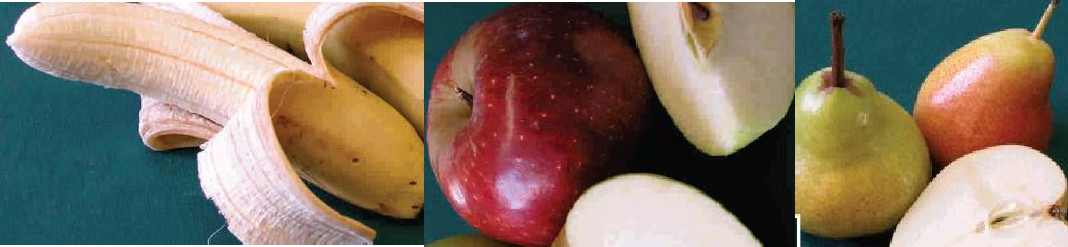

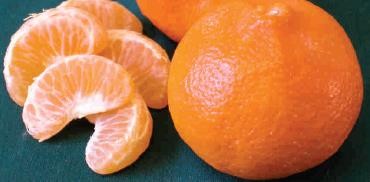

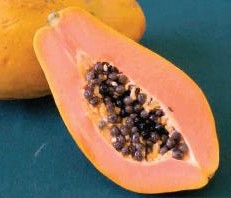

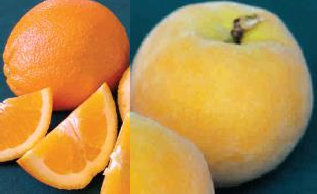

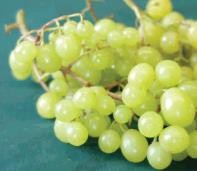

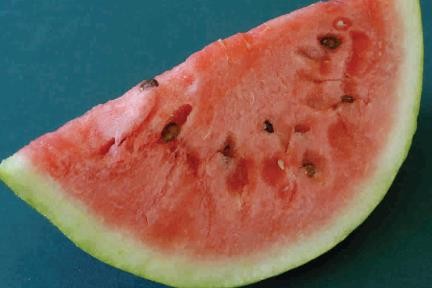

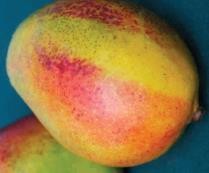


**1. Fruit**


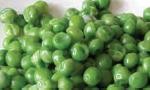

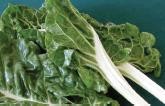

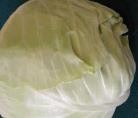

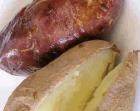

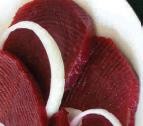

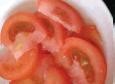

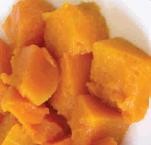

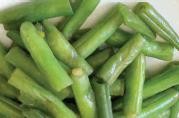

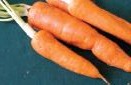

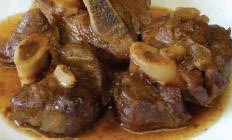

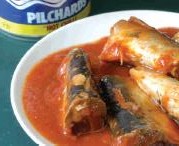

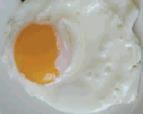

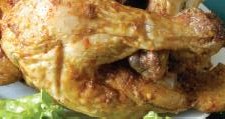

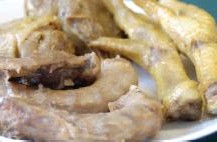

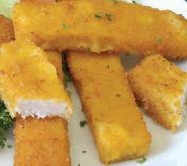

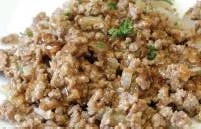

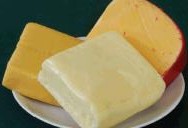

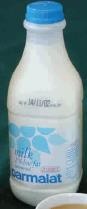

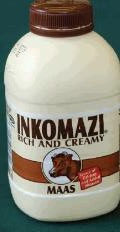

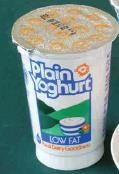


**4.**

**Milk, maas, yogurt cheese**

**3. Meat, chicken, fish or eggs**

Not polony or Russians or pies

**2. Vegetables**

5. Cold drinks


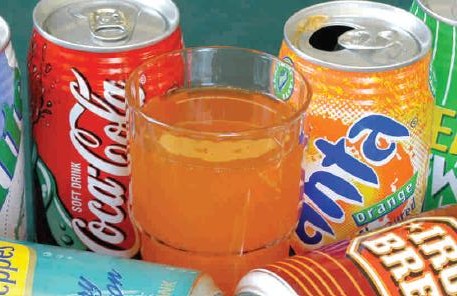

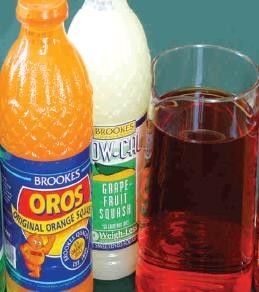

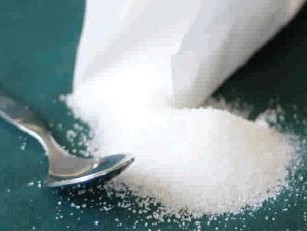

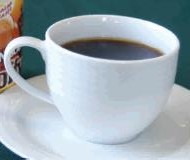

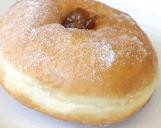

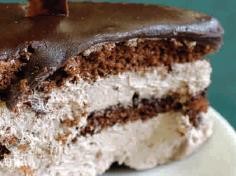

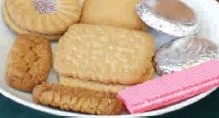

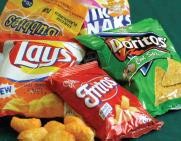

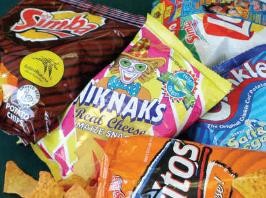

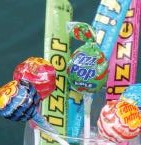

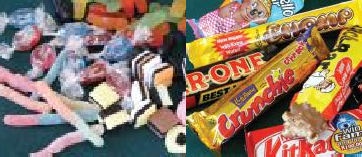

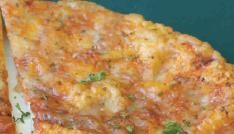

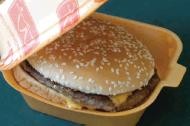

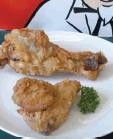

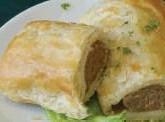


**10. Fast foods**

**9. Sweets, chocolates**

**8. Chips, cheese puffs, niknaks**

**7. Cakes, cookies**

**6. Sugar in tea and coffee**
