# Supplementary material for: Validation of a short food group questionnaire to determine intakes from healthy and unhealthy food groups in 5–9‐year‐old South African children
Source: J Hum Nutr Diet. 2023 Oct 5;37(1):234–45. doi: 10.1111/jhn.13249 (PMC10953415; doi:10.1111/jhn.13249)
Supplement: Supplementary file 2 — Supporting information. [file JHN-37-234-s001.docx]

**Appendix S2: A short food group questionnaire to determine intakes of healthy and unhealthy food groups.**

**Healthy and unhealthy food intake survey Participant nr: ……**

**Dear Parent or Caregiver**

How many times per week does your child take the following foods or drinks?

Please look at the pictures attached to this questionnaire to see examples of the 10 groups of foods and drinks that you must tick in the following list.

Please also remember to fill in **how many teaspoons of sugar** the child takes in his/her tea or coffee.

| **Food group** | **Frequency of intake per week** | | | | |
| --- | --- | --- | --- | --- | --- |
|  | 0 days | 1-2 days | 3-4 days | 5-6 days | 7 days |
| 1. Fruits (excluding canned fruit) |  |  |  |  |  |
| 1. Vegetables |  |  |  |  |  |
| 1. Meat, chicken, eggs or fish |  |  |  |  |  |
| 1. Milk, maas (inkhomazi), plain yogurt |  |  |  |  |  |
| 1. Cold drinks (fizzy drinks or cordials) |  |  |  |  |  |
| 1. Sugar in tea **(….teaspoons sugar/cup)** |  |  |  |  |  |
| 1. Cookies or cake |  |  |  |  |  |
| 1. Chips (Simba type) or cheese puffs |  |  |  |  |  |
| 1. Sweets or chocolates |  |  |  |  |  |
| 1. Fast foods (Fried chicken, Russians, Viennas, polony, hamburger, chips, pie, pizza) |  |  |  |  |  |
